# An Asylum Romance

**Published:** 1888-09-22

**Authors:** 


### AN ASYLUM ROMANCE.

### BEING THE TALE OF A TRAVELLER.

IT was in the autumn of a year which shall be nameless, that, oppressed with work, I sought for rest and relaxation in the quiet and solitude to be found in a retired village in the Midlands. Here I remained for some weeks, but did not gain that fillip to his exhausted energies without which Nature declined to rejuvenate him, or even to restore that normal amount of nervous energy, wanting which life is a burden, and the best work an impossibility. One cause of this failure was, no doubt, attributable to the ease with which I could be communicated with by those who were accustomed to rely upon his guidance and judgment in matters of business, with the natural consequence that this retirement into the country meant merely a change of residence and scene, but not cessation from work. With the prospect of a specially arduous period of hard brain work during the last two months of the year, it became necessary for him to try some other method of restoring his energies to their normal condition of vigorous health. Seeking for a remedy, it suddenly occurred to him that Holland would present many novel features of interest and diversion, and to that country, therefore, he decided to go, for the sufficient reason that there he would be less likely to meet crowds of tourists, and not unlikely to find many conditions of life well calculated to divert the thoughts to "fresh fields and pastures new."

It did not take long to put this idea into action, but one difficulty presented itself at the outset, which caused some unavoidable delay. Who should be selected as a companion for the journey? How often has this question been asked? It is, indeed, one which is only second in importance to that which all men, in the best sense and meaning of that word, have eventually to decide when the time arrives in their career to select a member of the opposite sex as a partner for life. There was this further obstacle in the present instance, that the period of the year was so advanced as to make it certain that nearly everybody had settled his plans if he had not already commenced to put them into execution. In the end the opportunity produced the man, who may be denominated for the purposes of the narrative, Dr. Careful. It is not necessary or desirable to detain the reader with any description of the circumstances of the journey, or of the many features of unique interest and instruction which the countries of Holland and Belgium present to the thoughtful and intelligent traveller. Suffice it to state that, in due course, we found ourselves located in one of the principal cities in the kingdom of Holland, where we took up our residence at an old-fashioned, but well-founded, hotel, which has a world-wide renown for its comfort and good management.

Hotel life in Holland partakes more of the character of family life, as we understand it in England, than such a condition probably presents in any other country in the world. First, there is that *persona grata*—the porter—who is usually a man of commanding presence, diplomatic caution, and excessive courtesy, possessing a full knowledge of everything concerning localities, modes of locomotion, places and persons which a long practice has shown him that most travellers desire to have. Speaking many tongues, he sees human nature under more varied circumstances than the majority of his fellow men, and for this reason he occupies in the hotel life of Holland a position of exceptional honour and power. He possesses a knowledge of character which makes his opinion respected, and, therefore, discretion forbids any attempt to argue, much less to quarrel, with so powerful a personage. Our porter was a representative specimen of his class, and he possessed the additional attribute of occupying the position of a trusted ally and confidant of the hotel proprietor, who was mainly guided by his counsel and opinion. The first introduction of the traveller to his fellows in a Dutch hotel is usually the table d'hôte dinner. The majority of our fellow-guests were English, and my right-hand neighbour was a lady, with whom I soon entered into an animated conversation. She presented the appearance of one who had seen much trouble, and whose lot was not, to say the least, a happy one. Still her features were well-formed, her powers of conversation good, her reading extensive, and her character evidently one which entitled her to possess the respect and affection of her intimate friends and near connections. Possessing a keen observation, she had noticed the conversation which had taken place between some of the members of the English Embassy and ourselves. This had evidently excited her curiosity, and we were, in consequence, discreetly, but somewhat persistently, cross-examined as to the circumstances which had brought us into the confines of the Dutch Republic. In self-defence, it was necessary to carry the war into the enemy's country, and, as a result, we ascertained, before dinner was over, that the gentleman on her left hand was her husband—a Dr. Richmond—who was the medical superintendent of one of the most extensive English asylums. The management of these institutions, and all that appertains to their welfare and efficiency, having long occupied the thoughts and attention of ourselves, we thus found a bond of mutual interest, which kept us friends throughout the rest of the tour.

It is strange, but true, that the Dutch possess a strong liking for what they denominate music, but to English ideas the word "noise" better describes the impression which the hearer receives by attending a performance of the Italian Opera, in the Dutch language, at a theatre in Holland. The Richmonds, Careful, and ourselves found this out by a visit to the Opera House one Saturday night, and we returned to the hotel more depressed than pleased by the performance. There was an absence of grace, and a fulness of form about the prima donna, combined with an extraordinary rotundity on the part of the ballet girls, and a want of histrionic ability in the case of the male performers, which left much to be desired. All this goes to show that novelties are not necessarily pleasing or beneficial.

It was our custom to write and read for some time before going to bed each evening; and after returning from the theatre on this occasion we remained so engaged until fully three a.m. About two o'clock in the morning I heard inharmonious sounds which my long residence in London and an imperfect attention to their precise meaning led me to attribute to the vocal efforts of the domestic cat, which pursues its ordinary avocations in the Dutch Republic with as much zeal and self-assertiveness as it does in the British Isles. No notice was therefore taken of the sounds in question, and the circumstance naturally failed to make any impression on our mind. On returning from church next morning Dr. Richmond found us out, and inquired whether we had heard any noises in the hotel in the night, as he knew we were in the habit of sitting up late. We gave him our impressions as described above. He then said, "It is reported, and generally believed in the hotel that the sounds were made by some lady who was being beaten by her husband as the result of a quarrel between them."

I merely laughed at the idea; congratulated myself that my wife was not with me, and suggested that he and the other married men should hold a council of war, spot the culprit, and deal with him summarily. He simply smiled, however, and passed on. Later in the day we had occasion to ask the porter whether Dr. Richmond was in the hotel, and as he seemed a little in doubt as to his identity we commenced to describe him. Thereupon he turned round, a little excitedly, and said, "I do know de gentleman vell enough; he is de English doctor who did beat his wife last night."

Upon this, we pointed out to the porter that such a charge was a very serious one to make, and wholly unjustifiable without evidence. He then said, "I know he beat his wife. He did get drunk; den he did quarrel with her, and den he did beat her *hard*, until she did scream out several times." We then felt bound, knowing the serious consequences which might result to Dr. Richmond if this story was allowed to remain uncontradicted, to proceed to a careful investigation of the circumstances on which the assertions of the porter rested, in the course of which we examined the proprietor of the hotel. The porter's story was, in effect, that the Richmonds had returned to the hotel from the theatre on the previous evening, Mrs. Richmond being accompanied by a young gentleman, who seemed to have some attractions for her, which Dr. Richmond resented by his manner and conduct. The Richmonds soon after retired to their room, and their young friend left the hotel, Dr. Richmond shortly afterwards coming down stairs and going out also. After the porter had gone to bed the night bell was rung, and Dr. Richmond was admitted. The porter said: "His manner was excited, but he was not den drunk, but he did get drunk after he did go to his room, where there were wine and brandy, both of which he did scatter all over de floor, making a nasty mess in several places." The porter, assuming a learned look, concluded the evidence against Dr. Richmond by summing up in these words:—

"You see, sir, he was not drunk when he did come home late last night, but he did get drunk upstairs before he got to bed. He did make himself very ill; he did beat his wife, and she did scream out loud."

We were naturally upset by this evidence, especially as the circumstance of Dr. Richmond having been the first person to draw our attention to the screaming might possibly mean that he wished us to understand that he, at any rate, was innocent, and knew nothing about the matter, except from hearsay. This view was confirmed by the circumstance, that Dr. Richmond had said, that he got out of bed on hearing the screaming, at his wife's earnest solicitation, and had partially dressed with the object of calling up one of his neighbours, and to go and see what was going on in the apartment from which the woman's screams proceeded. They had ceased however, and he therefore did not fulfil this intention; Fortunately we determined to go over the rooms on the same floor as that on which the Richmonds slept, and we found that there was a room immediately adjacent to it which had been occupied for three weeks by two German ladies, who were sisters. The landlord declared that they were very quiet and inoffensive people, and that it was impossible that the sounds could have proceeded from them. However, at our earnest solicitation, he consented to ask the ladies if either of them had made a noise on the night in question. On being appealed to, they stated that one them had had nightmare; and had hit out vigorously with her closed hand, which encountered the face of her sister, who thereupon awoke with a scream. This satisfied the landlord and the porter that they were labouring under a mistake, cleared the Richmonds from a most disagreeable charge; and caused the incident itself to assume an aspect more comic than tragic.

EPILOGUE.

At a dinner party soon after our return to England we met some friends of the Richmonds who told us the following facts. Dr. Richmond was engaged to his wife, who was a member of an influential county family but possessed of no private means of her own, for some long time before he became an asylum superintendent. The absence of sufficient means was the chief cause of the delay in their marriage, and this circumstance caused considerable sympathy to be created in the county for Mrs. Richmond. As a result of this, when the superintendentship of the asylum which Dr. Richmond now occupied fell vacant, everybody in the county exerted himself for her sake to secure him the appointment, and he was consequently elected. Imagine the disgust and indignation which followed, when, shortly afterwards, Dr. Richmond broke off his engagement, on the ground that his friends would not consent to the marriage. This indignation resulted in an action for breach of promise of marriage, and the jury found for the lady, with damages amounting to £1,500, and costs. Dr. Richmond had little or no money, his friends declined to help him, and the consequence was that he again proposed to the lady, was accepted, and they were married only a few weeks previous to the time of our meeting in Holland.

It is for the reader to decide from whence the screams in the Dutch hotel proceeded, and the author and cause of those screams. Had they, in short, a German or British origin? It is a delicate question, involving issues of serious moment to all concerned, and that intelligent jury, the British public, will, no doubt, in due course, arrive at a satisfactory judgment. At any rate, we feel that we did our duty to our countryman, and that, so far as the wily Dutchman is concerned, if inquiries should ever be made on the spot, he will doubtless declare that he satisfied himself that the suspicions which first entered his mind were unfounded and unjust, and that they were disposed of and dispersed by careful investigation at the time.

